# *Grateloupia tenuis* Wang et Luan sp. nov. (Halymeniaceae, Rhodophyta): A New Species from South China Sea Based on Morphological Observation and *rbc*L Gene Sequences Analysis

**DOI:** 10.1155/2013/560163

**Published:** 2013-12-23

**Authors:** Ling Yu, Hongwei Wang, Rixiao Luan

**Affiliations:** ^1^College of Life Sciences, Liaoning Normal University, Dalian 116029, China; ^2^Dalian Natural History Museum, Dalian 116023, China

## Abstract

*Grateloupia tenuis* Wang et Luan sp. nov. is a new species described from Lingshui, Hainan Province, South China Sea. Based on the external form and internal structure, combined with *rbc*L gene sequence analysis, *Grateloupia tenuis* is distinct from other *Grateloupia* species as follows: (1) thalli is slippery and cartilaginous in texture; possess fewer branches, relatively slight main axes, and two or three dichotomous branches; (2) cortex is 5-6 layers; medulla is solid when young, but hollow in old branches; reproductive structures are dispersed in main axes of thalli and lower portions of branchlets; exhibits *Grateloupia*-type auxiliary cell ampullae; (3) the four studied *G. tenuis * sequences were positioned in a large *Grateloupia* clade of Halymeniaceae, which included sister group generitype *G. filicina* with 68 bp differences; *G. tenuis* was determined to be a sister taxon to the *G. catenata*, *G. ramosissima*, *G. orientalis*, and *G. filiformis* subclade. The pairwise distances between *G. tenuis* and these species were 39 to 50 bp. The sequences of *G. tenuis* differed by 81–108 bp from the sequences of other samples in *Grateloupia*; there are 114–133 bp changes between *G. tenuis* and other genera of Halymeniaceae. In final analysis, we considered *Grateloupia tenuis* Wang et Luan sp. nov. to be a new species of genus *Grateloupia*.

## 1. Introduction

Red algae *Grateloupia *C. Agardh is a species-rich genus in the family Halymeniaceae, which not only exhibits highly diverse external morphology, but also is one of the genera in which species discrimination is more difficult. This genus is widely distributed in tropical and temperate coastal areas and includes more than 80 species worldwide and a total of 35 species in China [1–12]. The genus *Grateloupia *was originally built on the foundation of three species, namely, *G*. *ornata*, *G*. *hystrix,* and *G*. *filicina*, with *G*. *filicina* identified as the genus generitype [13].

Species morphology is significantly varied in *Grateloupia*, determining difficulties in species identification and controversy in species classification between algae taxonomists. Yendo [14] described* Grateloupia* with lomentaceous features as “catenate” and proposed *G. catenata *as a new species; however, Howe [15] regarded *Grateloupia *with lomentaceous characteristics as *G*.* filicina* (Lamouroux) C. Agardh var.* lomentaria* Howe. Okamura [16] opined that *G*. *filicina* var. *lomentaria *was a mature stage of *G*. *filicina* var. *porracea*, and integrated them into *G*. *filicina* var.* porracea *f. *lomentaria* (Howe) Okamura, with *G. catenata* being a synonym. Wang et al. [1] inspected type material of *G. catenata *Yendo and proposed to reinstate the Yendo name. *G. ramosissima *Okamura was discovered by Lin et al. [9], and its morphology resembles the algae researched in the present study. *G. orientalis* was described based on studies of cystocarp development and *rbc*L gene sequence analysis [9]. Zhang et al. [12] confirmed a new record of *G. orientalis* in the Hainan province of China through morphological observations, development of reproductive structures and molecular phylogenetic studies.

In our current research, the above species (*G. filicina, G. catenata, G. ramosissima *and* G. orientalis*) showed close relationships with *G. tenuis*; however, due to the particularly large differences in external and internal morphologies between *G. tenuis* and other known algae, this species has not been mentioned in previous reports. Based on these differences and analysis of ribulose-1,5-bisphosphate carboxylase/oxygenase (*rbc*L) gene sequences, we recommend that this alga is a new species of *Grateloupia* and should be defined as *Grateloupia tenuis* Wang et Luan sp. nov.

## 2. Materials and Methods

### 2.1. Morphological Analysis

Specimens were collected from the coast of Lingshui, Hainan Province, South China Sea (5 February 2009, leg. R. X. Luan; LNU20092087, LNU20092088, LNU20092089, and LNU20092090). Voucher herbarium specimens are reserved in the Herbarium of the College of Life Sciences, Liaoning Normal University, Dalian, China (LNU). We took *G*.* tenuis *(LNU20092088) as the holotype.

Morphological observations were made on algal specimens preserved in 10% seawater or pressed on herbarium sheets, and molecular analysis was conducted on samples desiccated in silica gel. Photographs of the holotype specimen were taken with a Canon EOS 650D (Canon, Japan). Hand sections were made by cryostat microtome, stained with 0.5% (w/v) cotton blue and discolored with 45% acetic acid. Photomicrographs were taken on an Olympus BH2 digital camera (Olympus Beijing Co. Ltd., China) mounted on a Nikon microscope (Nikon Corporation, Japan).

### 2.2. DNA Extraction and Phylogenetic Tree Construction

DNA samples of LNU20092087, LNU20092088, LNU20092089, and LNU20092090 were extracted using the DNeasy Plant Mini Kit (QIAGEN, Valencia, CA, Beijing). The procedures for PCR amplification and sequencing were executed as described previously [1]. The *rbc*L gene sequences of 32 extra relevant Halymeniaceae species and two additional related family (Gelidiaceae and Gracilariaceae) species were selected from GenBank for analysis and were involved in the alignment (Table 1). *Gelidiella ligulata*  Dawson and *Gracilaria tenuistipitata* Chang et Xia were treated as outgroups.

The *rbc*L sequences were aligned and compiled with Clustal X version 1.83 [21] for phylogenetic analysis. Phylogenetic tree construction and nucleotide differences analyses were conducted using MEGA 5.0. Maximum parsimony (MP), neighbor joining (NJ), and maximum likelihood (ML) were adopted to construct the phylogenetic tree. The MP analysis used heuristic searches for evaluating tree likelihoods, which was carried out with 1000 replicates, employed random addition sequence of taxa and used tree-bisection-reconnection (TBR) branch swapping [22]. The NJ analysis used the ratio test for estimation to seek optimal settings and ensure data dependability and was performed with Modeltest version 3.06 [23]. For ML analysis, a variety of cumulatively complex models of molecular evolution were assessed, as summarized by Litaker et al. [24] and Moncalvo et al. [21]. Bootstrap support values were calculated using 1000 samplings of the dataset [25] to estimate statistical reliability for MP, NJ, and ML methods.

## 3. Results

### 3.1. Taxonomic Descriptions

**Figure 1:**
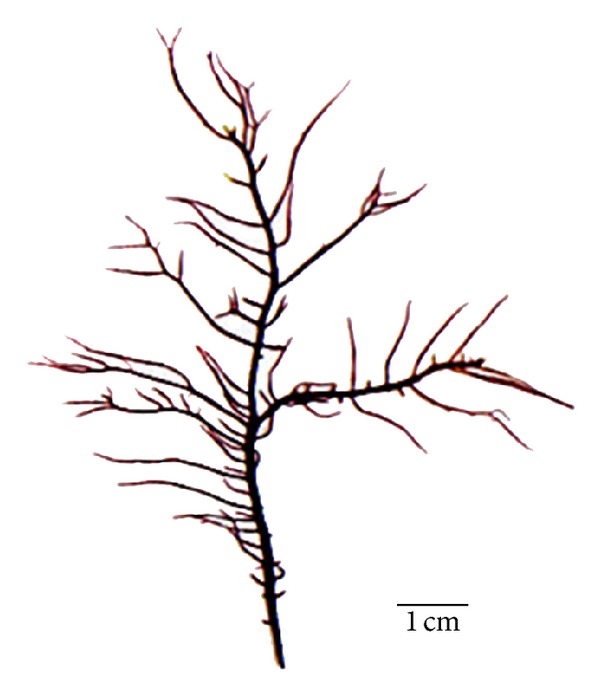
Holotype specimen of *Grateloupia tenuis* (female gametophyte collected from Lingshui, LNU20092088).

**Figure 2:**
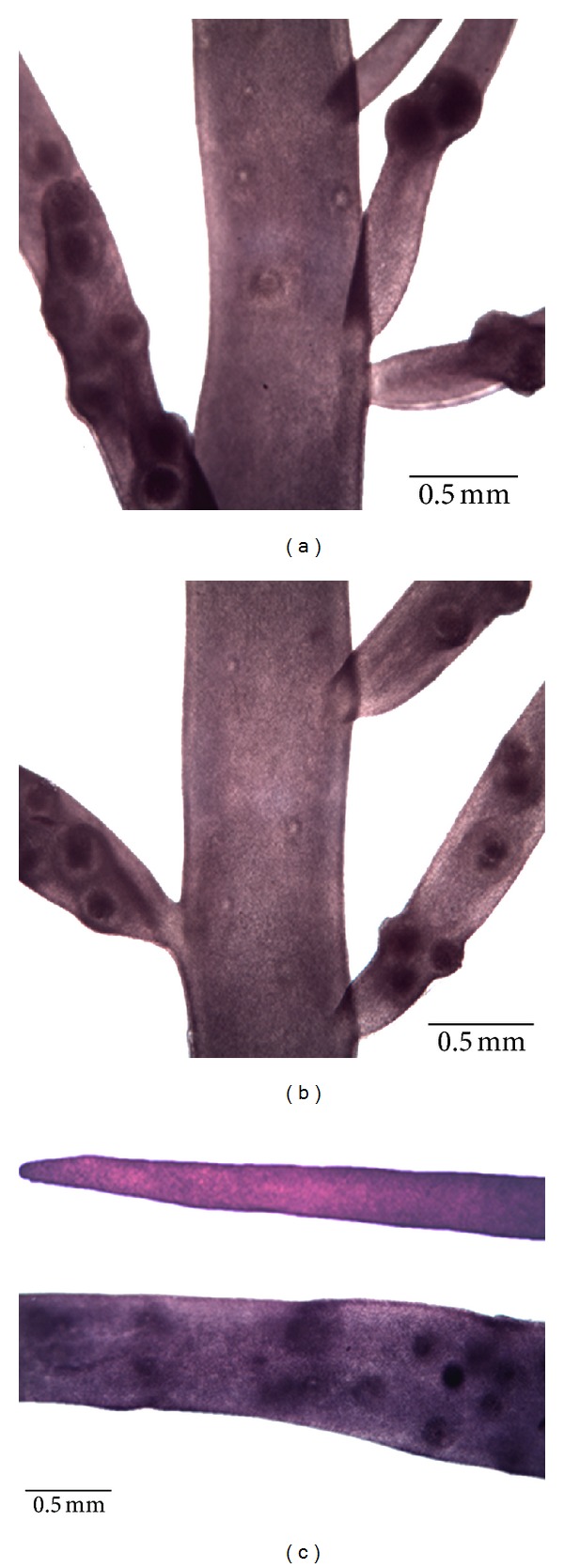
Branching characteristics and distribution of reproductive structures. (a) Second branches and distribution of cystocarp. (b) Alternation branches and distribution of cystocarp. (c) Cystocarp only dispersed in lower portion of branches.

Thallus simplex, linear, purplish red in color, cartilaginous and slippery in texture, 4–7 cm in height, main axes about 1 mm in width, branches about 0.2–0.6 mm in width, with fewer branches than other thalli (Figure 1). Main axes of thalli and branches relatively slight, erect axes cylindrical or subcylindrical and bearing alternation or second branches, twice or thrice dichotomously branched (Figures 2(a) and 2(b)). Cortex 5-6 layers, cells elliptic or polygonal and arranged densely; medulla solid with relatively intensive filaments when young, but hollow in the center in old branches. Gametophytes dioecious with reproductive structures dispersed in main axes of thalli and lower portion of branches, and cystocarps of main axes significantly fewer than branches (Figures 2(a)–2(c)). Carposporangium developed from gonimoblasts cells, profoundly immersed inside medulla and revolved by branched ampullar filaments. Mature cystocarps 90–110 *μ*m in diameter. Spermatangium shaped from outermost cortex cells of male gametophytes. Mature tetrasporangium cruciately partitioned, 10–16 *μ*m long, 6–8 *μ*m in diameter, and inserted in outer cortical cells.

Consider the following.

 Etymology; “*tenuis*” refers to the morphology of the thallus.
 Holotype; appointed here is a female specimen (LNU20092088 Figure 1).
 Type locality; Lingshui, Hainan Province, China, 5 Feb. 2009, collected by R. X. Luan.
 Habitat and seasonality; plant collections were seasonal from January to March, and were attached on rocky reefs or stony marsh of coastal intertidal zone.
 Distribution; currently only known in Lingshui, Hainan Province, China (E109.9°, N18.4°).

### 3.2. Vegetative and Reproductive Structures

**Figure 3:**
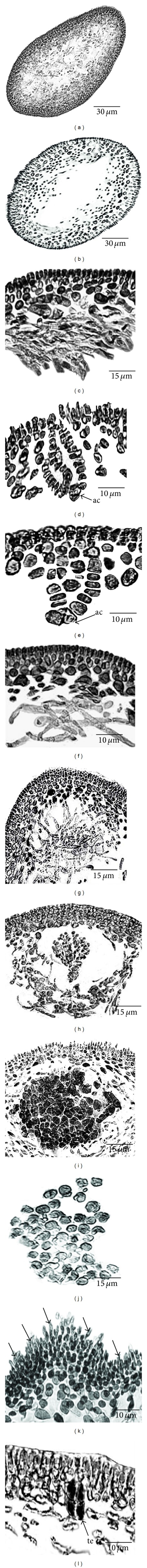
Vegetative and reproductive structures of *Grateloupia tenuis*. (a) Cross-section of thallus displaying cortex and solid medulla when young. (b) Cortex and hollow medulla when old. (c) Internal structure of branches displaying arrangement and layers between cortex and medulla. ((d), (e)) Structure of carpogonial branch ampulla and auxiliary cell ampulla. ((f)–(i)) Successive periods of cystocarp development. (f) Initial phase of cystocarp formation. (g) Middle phase of cystocarp formation. (h) Cystocarp development. (i) Advanced stage of cystocarp development and mature cystocarps. (j) Carpospores. (k) Construction of spermatangium. (l) Mature tetrasporangium (te).

Cross-section of the thallus showed a densely arranged cortex and solid medulla with dense filaments when young, but hollow in the center in old branches (Figures 3(a) and 3(b)). The 5-6 layers of the cortex consisted of oval outer layers cells arranged in neat rows and three to four inner layers of elliptic or rounded cells; the medulla was composed of slender medullary filaments 11–17 *μ*m long and 1-2 *μ*m wide, which exhibited irregular distribution (Figure 3(c)). The thallus had *Grateloupia*-type auxiliary cell ampullae. Auxiliary cells (ac) and carpogonial branches were produced in independent ampulla initiated from inner cortical cells. Two-celled carpogonial branches existed in each carpogonial branch ampulla and contained a terminal carpogonium and hypogynous cell (Figure 3(d)). The auxiliary cell ampullae were narrowly bottle-shaped and comprised of two or three secondary filaments. The mature auxiliary cell (ac) was elliptical in shape and obviously larger than other ampullary cells, and was located at the basal part of the ampulla (Figure 3(e)). Successive stages of the cystocarp development are displayed in Figures 3(f)–3(i). Terminal cells of the gonimoblast filaments gradually matured (Figure 3(f)). Maturing carposporangia developed from gonimoblasts cells, and were surrounded by branched ampullar filaments (Figure 3(g)). As cystocarps proceeded with development, they became increasingly larger and more deeply embedded in the medulla (Figure 3(h)). Mature cystocarps were spherical or ellipsoidal and 90–110 *μ*m in diameter (Figure 3(i)). The mature cystocarp released carpospores (Figure 3(j)). Spermatangia of the male gametophytes were shaped from the outermost cortex cells (Figure 3(k)). Mature tetrasporangia formed from the sporophyte cortical cells were cruciately partitioned, 10–16 *μ*m long, and 6–8 *μ*m in diameter (Figure 3(l)).

### 3.3. Molecular Analysis

In the phylogenetic tree, we compared four *G*. *tenuis rbc*L gene sequences with a total of 34 extra *rbc*L sequences from taxa comprising 28 species of *Grateloupia*, with four species from other genera (*Halymenia* C. Agardh and *Polyopes* J. Agardh) in Halymeniaceae and two species from Gelidiaceae and Gracilariaceae treated as outgroups, which were selected from GenBank for analyses (Table 1). The *rbc*L sequences of the four Lingshui samples were uniform. The *rbc*L sequence alignment consisted of 1322 base pairs (bp), but since many *rbc*L sequences were incomplete at the 5′ and 3′ ends, the first 67 bp and last 15 bp were excluded from analyses.

**Figure 4:**
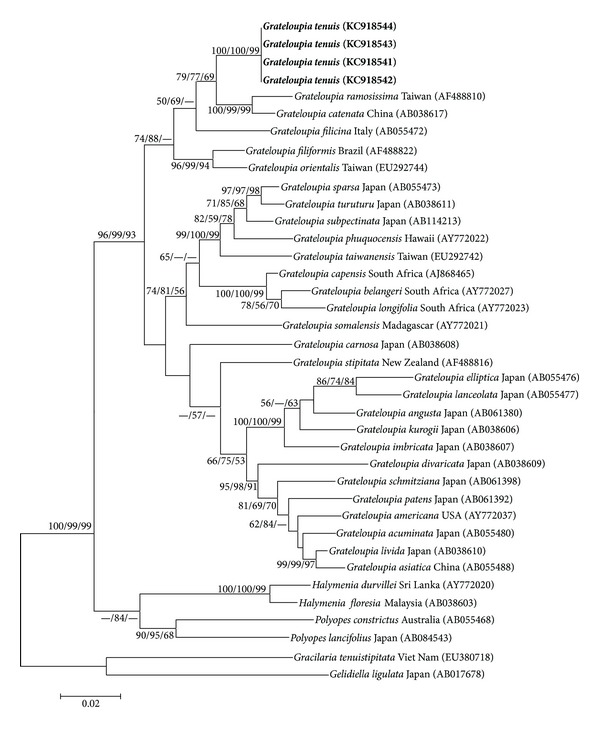
Maximum likelihood tree showing phylogenetic relationships of *G*.* tenuis* and relevant genera within *Grateloupia*, inferred from partial *rbc*L gene sequences (1240 bp). *Gelidiella ligulata* and *Gracilaria tenuistipitata* were treated as outgroups. Numbers at internal nodes are bootstrap proportion values (1000 replicates) for ML, NJ, and MP, which only show above 50% bootstrap support. Branch lengths are proportional to the amount of sequence change. Boldface displays new species depicted in this research.

The maximum likelihood phylogenetic tree (Figure 4) was obtained through the NJ, MP, and ML analysis. The species aggregate of the ML phylogenetic tree was divided into three main clades with high bootstrap support values and included a large *Grateloupia* clade; a *Halymenia*/*Polyopes *clade, which were all within Halymeniaceae; and a *Gelidiella*/*Gracilaria *clade, which were in Gelidiaceae and Gracilariaceae, respectively. The four *G*.* tenuis* samples and the 28 kinds of *Grateloupia* genera formed together into one large clade. The *Grateloupia* clade was divided into a small and large clade, and *G*.* tenuis *formed a monophyletic group within the small clade.

The *rbc*L analyses of pairwise base differences between *G*.* tenuis *and generitype *G*. *filicina* were 68 bp changes (5.72%). Sequences among *G*.* tenuis* and *G*. *catenata* and *G*. *ramosissima* differed by 39 bp (3.22%) and 48 bp (3.98%). There were 45 bp (3.73%) and 50 bp (4.17%) differences between *G*.* tenuis* and *G*. *orientalis* and *G. filiformis*, respectively. Sequences between *G*.* tenuis* and other samples in *Grateloupia *ranged from 81–108 bp changes (6.88%–9.34%). Divergence between *G. tenuis* specimens and other genera of Halymeniaceae ranged from 114–133 bp differences (9.86%–11.66%). When compared to outgroups, the *G*.* tenuis* specimens exhibited 190 bp (17.17%) and 196 bp (17.75%) differences from *Gracilaria tenuistipitata *(Gracilariaceae) and* Gelidiella ligulata *(Gelidiaceae), respectively.

## 4. Discussion

The family Halymeniaceae exhibits high species richness, especially within *Grateloupia*, which makes taxonomic species identification difficult. Nevertheless, the formation of auxiliary cell ampullae is a remarkable characteristic for distinguishing genera within Halymeniaceae [2, 3, 6, 26].

The newly described *G*. *tenuis *species is morphologically similar to some species, especially *G. catenata*.  Table 2 shows a comparison in morphological features among *G*.* tenuis* and the closely related species *G*. *catenata*, *G*.* ramosissima*,* G*. *orientalis*, *G*. *filicina*. It was easy to differentiate *G*.* tenuis *from* G*. *catenata* by its significantly smaller size (4–7 cm high compared to 35 cm high, resp.) and by its dispersed reproductive structures in the main axes of thalli and lower portions of branchlets compared to the scattered reproductive structures over the whole thallus in *G*. *catenata*. It was possible to separate *G*.* tenuis *from generitype *G*. *filicina* by its slippery and cartilaginous texture compared with the mucilaginous and hard texture of *G*. *filicina*, and by its dichotomous branches compared with pinnate branchlets. Distinction with *G*. *ramosissima *and *G*.* orientalis* showed that they had abundant branches. *G*. *tenuis *also had representative *Grateloupia*-type auxiliary cell ampullae, which demonstrated that it was a new species of the family Halymeniaceae and pertained to the genus *Grateloupia*.

The* rbc*L sequence data also strongly supported *G*.* tenuis *as a new species. In the ML phylogenetic tree (Figure 4), *G. tenuis* specimens were embedded in the large clade of *Grateloupia *and clustered into a single monophyletic group. The small clade of *Grateloupia* included *G*. *catenata*, *G*. *ramosissima*, *G*. *filiformis*, and *G*. *orientalis* from China, and generitype *G*. *filicina* from Italy. The *G*. *catenata*/*G*. *ramosissima *subclade was the most closely related sister taxon to *G*. *tenuis* and the most similar in appearance. In addition, *G. tenuis *produced a high bootstrap support value with the *G*.* catenata/G*.* ramosissima *subclade. The generitype species, *G*. *filicina*, belonged to the sister position of *G*. *tenuis*, which strongly supported that *G*. *tenuis* phylogenetically approached *G*. *filicina*. Moreover, *G*. *orientalis*, and *G*. *filiformis *formed a sister clade with *G*.* tenuis*. All *Grateloupia* species were phylogenetically different to the two clades, especially *Gelidiella ligulata *and* Gracilaria tenuistipitata *serving as outgroups.

## 5. Conclusions

From morphological observation and *rbc*L gene sequence analysis, we concluded that the studied specimen was a new species of genus *Grateloupia*, defined as *Grateloupia tenuis* Wang et Luan sp. nov. Currently, due to algae species diversity, traditional taxonomic methods are not the most effective way to identify species. With the rapid development of molecular biology and gene sequencing technology; however, taxonomists are increasingly using combined morphological observation with DNA barcoding for algae classification. In recent years, human impact on marine ecosystems has increased the urgent need to conserve aquatic resources, and accurate species identification is a basic prerequisite in helping protect marine algae.

## Figures and Tables

**Table 1:** List of species for *rbc*L sequences analysis, collection data, and accession numbers in GenBank.

Species	Collection data (location, site, and reference)	GenBank accession numbers
*Grateloupia tenuis* Wang et Luan	Lingshui, Hainan Province, China (LNU20092087)	KC918541
*Grateloupia tenuis* Wang et Luan	Lingshui, Hainan Province, China (LNU20092088)	KC918542
*Grateloupia tenuis* Wang et Luan	Lingshui, Hainan Province, China (LNU20092089)	KC918543
*Grateloupia tenuis* Wang et Luan	Lingshui, Hainan Province, China (LNU20092090)	KC918544
*G*.* catenata* Yendo	Shimiao, Dalian, Liaoning Province, China (Wang et al., 2000) [1]	AB038617
*G*.* ramosissima* Okamura	Ho Ping Island, Keel Nug, Taiwan Province, China (Gavio and Fredericq, 2002) [4]	AF488810
*G*.* filicina* (Lamouroux) C. Agardh	Livorno, Italy (Wang et al., 2000) [1]	AB055472
*G*.* orientalis* Lin et Liang	Linyuan, southwestern Taiwan Province, China (Lin et al., 2008) [9]	EU292744
*G*.* filiformis* Kutzing	Marataizes, Espiritu Santu, Brazil (Gavio and Fredericq, 2002) [4]	AF488822
*G*.* carnosa* Yamada et Segawa	Oryuzako, Miyazaki Prefecture, Japan (Wang et al., 2000) [1]	AB038608
*G*.* stipitata* J. Agardh	Lee Bay, Stewart Island, New Zealand (Gavio and Fredericq, 2002) [4]	AF488816
*G*.* acuminata* Holmes	Katase, Fujisawa, Kanagawa Prefecture, Japan (Kawaguchi et al, 2001) [3]	AB055480
*G*.* americana* Kawaguchi et Wang	Pigeon Point, San Matio County, California, USA (De Clerck et al., 2005) [7]	AY772037
*G*.* asiatica* Kawaguchi et Wang	Qingdao, Shandong Province, China (Kawaguchi et al., 2001) [3]	AB055488
*G*.* livida* (Harvey) Yamada	Izu-misaki, Miyake Island, Tokyo, Japan (Wang et al., 2000) [1]	AB038610
*G*.* patens *(Okamura) Kawaguchi et Wang	Oohara, Chiba Prefecture, Japan (Wang et al., 2001) [2]	AB061392
*G*.* divaricata* Okamura	Oshoro, Hokkaido, Japan (Wang et al., 2000) [1]	AB038609
*G*.* schmitziana* (Okamura) Kawaguchi et Wang	Shichirigahama, Kamakura, Kanagawa, Japan (Wang et al., 2000) [1]	AB061398
*G*.* lanceolata* (Okamura) Kawaguchi	Shikanoshima, Fukuoka, Japan (Kawaguchi et al., 2001) [3]	AB055477
*G*.* elliptica* Homles	Goshikinohama, Usa, Tosa, Kochi Prefecture, Japan (Kawaguchi et al., 2001) [3]	AB055476
*G*.* kurogii* Kawaguchi	Saikai-bashi, Nagasaki Prefecture, Japan (Wang et al., 2001) [2]	AB038606
*G. phuquocensis* Tanaka et Pham-Hoang	Kaalawai, Oahu, Hawaii (De Clerck et al., 2005) [6]	AY772022
*G. sparsa* (Okamura) Chiang	Oohara, Chiba Prefecture, Japan (Wang et al., 2000) [1]	AB055473
*G*.* imbricata* Holmes	Tsuyazaki, Fukuoka Prefecture, Japan (Wang et al., 2000) [1]	AB038607
*G*.* longifolia* Kylin	Yzerfonteyn, Western Cape Province, South Africa (De Clerck et al., 2005) [6]	AY772023
*G*.* belangeri* (Bory de Saint-Vincent) Setchell et Gardner	Platboom, Western Cape Province (De Clerck et al., 2005) [6]	AY772027
*G*.* angusta* (Okamura) Kawaguchi et Wang	Miyanoura, Hirado Island, Nagasaki Prefecture, Japan (Wang et al., 2001) [2]	AB061380
*G*.* capensis* De Clerck	South Africa (De Clerck et al., 2005) [6]	AJ868465
*G*.* somalensis* Hauck	Plage de Monseigneur, Fort Dauphin, Madagascar (De Clerck et al., 2005) [7]	AY772021
*G*. *taiwanensis* Lin et Liang	Northeastern and southern Taiwan (Lin et al., 2008) [9]	EU292742
*G*.* subpectinata* Holmes	Irago-misaki, Atsumi, Aichi, Japan (Faye et al., 2004) [5]	AB114213
*G*.* turuturu* Yamada	Muroran, Hokkaido, Japan, South Africa (Wang et al., 2000) [1]	AB038611
*Halymenia durvillei *Bory	Beruwela, Sri Lanka (De Clerck et al. 2005) [6]	AY772020
*Halymenia floresia *(Clemente) C. Agardh	Pulau Rebak Besar, Langkawi, Kedah, Malaysia (Wang et al., 2000) [1]	AB038603
*Polyopes constrictus* (Turner) J. Agardh	Point Lonsdale, Victoria, Australia (Kawaguchi et al., 2001) [3]	AB055468
*Polyopes lancifolius *(Harvey) Kawaguichi et Wang	Inoshiri, Usa, Tosa, Kochi, Japan (Kawaguchi et al., 2002) [17]	AB084543
*Gelidiella ligulata* Dawson	Miyake Island, Tokyo, Japan (Shimada et al., 1998) [18]	AB017678
*Gracilaria tenuistipitata* Chang et Xia	Viet Nam (Gurgel et al., 2008) [19]	EU380718

**Table 2:** Comparison of morphological characteristics between *Grateloupia  tenuis* and other closely related species.

Morphological characteristics	*G*.* tenuis *	*G*.* filicina *	*G*.* catenata *	*G*.* ramosissima *	*G*.* orientalis *
Thallus habit	Fewer branches and main axes relatively slight; two or three dichotomous branches; 4–7 cm high	Erect axes; several terete to flattened blades with irregularly pinnate branchlets; 9–12 cm high	Erect axes; terete to compressed branches and tapering towards tapex; up to 35 cm high	Erect axes; linear to several lanceolate blades; 13–22 cm high, up to 1 mm wide	Thalli terete to slightly compressed branches bearing irregularly pinnate branches; 10–16 cm high

Texture	Slippery and cartilaginous	Mucilaginous and hard	Gelatinous	Cartilaginous	Gelatinous to cartilaginous

Cortex (C)	C: 5-6 layers	C: 5–8 layers	C: 6–14 layers	C: 8-9 layers	C: 6–9 layers

Medullary	Hollow	Solid	Hollow	Solid	Hollow

Location of reproductive structures	Main axes and lower portions of branches	Proliferations and upper portions of thallus	Whole thallus	Entire thalli	Whole thalli except the basal parts

Distribution	Lingshui, Hainan province, China	Italy, France, Spain	China, Japan, Korea	Japan, China, Korea, Vietnam, Philippine	Taiwan

Reference	This study	Kawaguchi et al., 2001 [3]	Wang et al., 2000 [1] Lee et al., 2009 [20]	Lin et al., 2008 [9]	Lin et al., 2008 [9] Zhang et al., 2012 [12]
